# Red Wine Age Estimation by the Alteration of Its Color Parameters: Fourier Transform Infrared Spectroscopy as a Tool to Monitor Wine Maturation Time

**DOI:** 10.1155/2017/5767613

**Published:** 2017-11-01

**Authors:** M. Basalekou, C. Pappas, Y. Kotseridis, P. A. Tarantilis, E. Kontaxakis, S. Kallithraka

**Affiliations:** ^1^Laboratory of Oenology, Department of Food Science & Human Nutrition, Agricultural University of Athens, 75 Iera Odos, 11855 Athens, Greece; ^2^Laboratory of Chemistry, Department of Food Science & Human Nutrition, Agricultural University of Athens, 75 Iera Odos, 11855 Athens, Greece; ^3^Department of Agriculture, School of Agriculture, Food and Nutrition, Technological Educational Institute of Crete, Estavromenos, 71004 Heraklion, Greece

## Abstract

Color, phenolic content, and chemical age values of red wines made from Cretan grape varieties (Kotsifali, Mandilari) were evaluated over nine months of maturation in different containers for two vintages. The wines differed greatly on their anthocyanin profiles. Mid-IR spectra were also recorded with the use of a Fourier Transform Infrared Spectrophotometer in ZnSe disk mode. Analysis of Variance was used to explore the parameter's dependency on time. Determination models were developed for the chemical age indexes using Partial Least Squares (PLS) (TQ Analyst software) considering the spectral region 1830–1500 cm^−1^. The correlation coefficients (*r*) for chemical age index i were 0.86 for Kotsifali (Root Mean Square Error of Calibration (RMSEC) = 0.067, Root Mean Square Error of Prediction (RMSEP) = 0,115, and Root Mean Square Error of Validation (RMSECV) = 0.164) and 0.90 for Mandilari (RMSEC = 0.050, RMSEP = 0.040, and RMSECV = 0.089). For chemical age index ii the correlation coefficients (*r*) were 0.86 and 0.97 for Kotsifali (RMSEC 0.044, RMSEP = 0.087, and RMSECV = 0.214) and Mandilari (RMSEC = 0.024, RMSEP = 0.033, and RMSECV = 0.078), respectively. The proposed method is simpler, less time consuming, and more economical and does not require chemical reagents.

## 1. Introduction

Wine age is a parameter of great importance as it is linked with wine quality. However, there is no direct way to measure it. Most information related to age is derived from the evolution of the wine's organoleptic characteristics, such as color, mouth feel, and aroma which depend on the phenolic and volatile content of the wine.

Color in particular is an indicator of the interactions between phenolic compounds, such as the replacement of monomer forms by polymers which are time dependent reactions. Young wines' color depends on the concentration of free anthocyanins, which on the wine's pH are on their colored form (positively charged flavylium). As monomers, they are highly susceptible to changes in the medium's environment, such as pH and SO_2_ changes [1]. During ageing, while monomers are replaced by polymers, color changes and stabilization occur. For example, color density is reduced as a result of oxidation but also due to changes in the structure of anthocyanin-tannin polymers and precipitation of some of the polymers formed. Moreover, the purple hue of young red wines is replaced by brickish red to tawny red hues. Those changes in color can be observed with the naked eye, but also easily using a spectrophotometer by measuring color parameters such as intensity and hue [2]. The transformation of monomeric pigments to more stable polymeric forms is well documented in research [3, 4]. The evolution of the physicochemical characteristics of wines occurs primarily during wine maturation and ageing, as phenolic compounds participate in numerous chemical reactions such as copigmentation and polymerization. One of them is the gradual formation of condensed pigments between free anthocyanins and colorless phenols present in grapes. These compounds can be formed either in the presence or in the absence of oxygen. When ageing is oxidative, that is, barrel ageing or vat ageing with high levels of aeration, the anthocyanins and tannins react also with acetaldehyde which is formed as a result of ethanol oxidation, to form pigments of various structures. These reactions are catalysed by ellagitannins, phenols that are extracted from the barrel [5]. The polymeric pigments can be further classified into two categories: long polymeric pigments (LPP) and short polymeric (SPP) pigments. LPP have a polymer length greater than three and can be precipitated with protein while SPP are shorter polymers that do not precipitate with proteins [6]. Copigmentation refers to the self-association of anthocyanins or their association with certain phenolic monomers and results in color stabilization due to hyperchromic and bathochromic shifts in the visible absorbance [7].

Monitoring the evolution of anthocyanins can be a demanding task in terms of analytical methods. For a time efficient and in-depth analysis of the anthocyanin forms over time, without the use of specified instruments, Somers introduced in 1977 the concept of “chemical age.” Chemical age is defined as the extent to which monomeric anthocyanins have been displaced by polymeric pigment forms [8]. Indices of chemical age are the indexes “chemical age i” and “chemical age ii,” which are derived from two spectral ratios. Both refer to the extent to which pigments have become less susceptible to the changes of pH and to SO_2_ bleaching and changes to which free anthocyanins are more susceptible as less stable. The sample absorbance at 520 nm is due to the free forms of anthocyanins, copigmented anthocyanins, and polymeric pigments. Absorbance is measured after pH adjustment or the addition of bisulfite solution or acetaldehyde to separate total anthocyanins and polymeric pigments. Lowering the pH of the wine allows the determination of free anthocyanins and polymeric pigments; acetaldehyde removes the bleaching effect of any SO_2_ present in the wine and the addition of bisulfite reveals the degree to which the color is due to the polymeric anthocyanin forms [9]. This UV-Vis based method commonly used in the wineries requires multiple step sample processing and measurements.

Given the importance of wine color, an indirect method able to determine the color parameters in red wines with reasonable accuracy and ease of use would be of great value to wine industry. Infrared spectroscopic methods have the advantage of being nondestructive and fast and require small sample quantities. When combined with multivariate data analysis, such as Partial Least Squares (PLS) regression analysis, they are suitable for correlating the spectral response of a sample with its chemical composition [10]. The application of both near- and mid-infrared spectroscopic methods to grape and wine analysis has become a valuable alternative to the slow and destructive analytical procedures which are routinely used as the classic UV-Vis and chromatographic techniques [11–16] or even for monitoring wine ageing in barrels [17].

The first applications of FT-IR for the quantification of total anthocyanins in wine were carried out by Versari et al. [18]. They also determined LPP, SPP, total wine color, and copigmented anthocyanins. The best results were obtained using vector normalization and no-preprocessing of spectral data. Later studies using direct orthogonal signal correction preprocessing have improved the prediction of polymeric pigments in red wines by FT-IR spectroscopy compared to the row data [19]. Picque et al. [20] also used FT-IR spectroscopy to build a predictive model that was able to quantify anthocyanins in red grape extracts. Anthocyanin prediction was improved if a separate calibration model was calculated for each geographical region. The best prediction for monitoring anthocyanins in red grapes using FT-IR was presented by Fragoso et al. [12]. They obtained a valid regression model for prediction of total anthocyanins working in the region of 979–2989 cm^−1^.

Individual anthocyanin concentration can also be predicted using FT-IR [21] in young red wines. Recently, Rasines-Perea et al. [22] determined twelve individual anthocyanins in red grape musts using FT-IR and PLS. However, in both the above studies there was a need to employ correction factors to improve the prediction due to systematic errors.

There exist a number of important studies concerning the changes in phenolic content and color parameters of red wines during ageing [23–25]. However, despite the importance of visual appearance to the final wine quality, there is not much published data concerning the correlation of color parameters with wine age. It was thus of interest to determine the alteration of selected color parameters of red wines during short maturation periods and to evaluate the overall changes with respect to their age.

Moreover, it was of interest to explore the suitability of FT-IR spectroscopy as a simple, less time consuming, and more economical technique to monitor wine chemical age which is of high technological importance.

## 2. Materials and Methods

### 2.1. Wines and Containers

The wines used were made of two major red grape varieties of Crete, Kotsifali and Mandilari, which differ greatly in their anthocyanic and tannic content. Kotsifali produces wines low in color and relatively high in alcohol with smooth tannins, whereas Mandilari produces age worthy wines with deep red color. All wines were vinified following the protocol for classical red winemaking and received the same SO_2_ additions. No tartaric acid additions were made as pH values for all wines were satisfactory (pH 3.4–3.5). For the maturation process, different types of containers were used (tank, tank with French oak chips and barrels made of French oak, American oak, Acacia, and Chestnut) and samples were taken from each container every three months over the period of nine months for two consecutive vintages (2012 and 2013) (12 months of contact only for the 2013 vintage), resulting in 12 red wine samples for each vintage's trimester (Table 1).

### 2.2. Spectrophotometric Analyses

Total anthocyanins (TA), degree of ionisation of anthocyanins (ID), color density (CD), color density corrected for SO_2_ (CDS), SO_2_ resistant pigments (SRP), and chemical ages i and ii were determined according to the modified Somers assay as described by Mercurio et al. [1]. According to the method, prior to analysis pH and alcohol content of all wine samples were standardized to 3.4 and 12% v/v, respectively, with the use of a buffer solution. After that, the wines are treated with excess SO_2_, excess acetaldehyde, and hydrochloric acid. The absorbance of the samples is read in four steps: first, the absorbance of wine in its original state is read at 420 and 520 nm (*A*_420  buffer_, *A*_520  buffer_); then the second reading is after the addition of excess SO2 (*A*_520  sulfite_). This reading allows the measurement of color resulting from SO2-resistant pigments. Subsequently, the absorbance of wine samples treated with excess acetaldehyde is read (*A*_520  acetal_), to estimate the anthocyanins which are colored at wine pH. Finally, the absorbance of wine diluted with hydrochloric acid is read (*A*_520  HCl_). This treatment lowers the pH of wine and this way all anthocyanins are converted into their colored forms.

*Calculations*

  Total anthocyanins (mg/L): 20 × [(50 × *A*_520  HCl_)−(1.6667 × (10 × *A*_520  sulfite_)]).
  Degree of ionisation of anthocyanins (%): 100 × [(10 × *A*_520  buffer_)−(10 × *A*_520  sulfite_)]/[(50 × *A*_520  HCL_) − 1.6667 × (10 × *A*_520  sulfite_)].
  Color density (au): (*A*_420  buffer_ + *A*_520  buffer_) × 10.
  Color density corrected for SO_2_ (au): (*A*_420  acetal_ + *A*_520  acetal_) × 10.
  SO_2_ resistant pigments (au): *A*_520  sulfite_ × 10.
  Chemical age i (no units): *A*_520  sulfite_/*A*_520  acetal_.
  Chemical age ii (no units): *A*_520  sulfite_/(5 × *A*_520  HCl_).

Both indexes are close to zero in wines right after fermentation is completed [8].

Color intensity (CI) and hue (h) were determined according to the method proposed by Glories [26] and were calculated after measuring the absorbance at 420, 520, and 620 nm as follows: CI = *A*_420_ + *A*_520_ + *A*_620_ and h = *A*_420_/*A*_520_.

All absorbance measurements were performed on a Hitachi U-2000 spectrophotometer.

### 2.3. FT-IR

Mid-IR spectra of all samples were collected with the use of a Fourier Transform Infrared Spectrophotometer (Thermo Nicolet 6700 FT-IR by Thermo Electron Corporation, MA, USA) equipped with a deuterated triglycine sulfate (DTGS) detector, following the procedure described by Basalekou et al. [17]. All samples' spectra were recorded in the region 4000–500 cm^−1^.

### 2.4. Statistical Analysis

Principal Component Analysis (PCA) and Analysis of Variance (ANOVA) were performed to plot differences between samples and to examine each variable's (vintage, time, and container) effect on wine color compounds. The statistical program used for these analyses was JMP v.11. Determination models were also developed using Partial Least Squares (PLS) and the FT-IR's built-in software TQ Analyst. PLS is a quantitative analysis technique. The PLS algorithm examines the specified region of the calibration spectra to determine areas that vary statistically as a function of component concentration. The calibration model is then developed using spectral and concentration information. The spectral region considered for the statistical analysis was 1830–1500 cm^−1^.

## 3. Results and Discussion

### 3.1. Chemical Analyses

Chemical analyses highlighted the differences in the phenolic content of Kotsifali and Mandilari wines. For Kotsifali, the maximum initial average concentration of anthocyanins (i.e., after fermentation completion) was 145.4 mg/L while for Mandilari it was 322.6 mg/L g/L (vintage, 2013). Mandilari wines are characterized as being rich in anthocyanins and tannins, presenting a deep red color and a quite astringent palate. Their high tannic and anthocyanin concentration favours the formation of more anthocyanin-tannin polymers.

The anthocyanin concentration of Kotsifali and Mandilari wines decreased threefold over the nine-month period of maturation for both vintages and in all containers (Table 2). Indeed, anthocyanins are unstable molecules and they are incorporated into the tannin structure, forming pigmented polymers. The decrease in anthocyanin concentrations is also associated with the formation of more stable pigments such as pyranoanthocyanins as well as the degradation of anthocyanins [27]. These results are in agreement with the results obtained by Kallithraka et al. [28]. The method used for anthocyanin determination is based on the effect of pH on anthocyanin structure. Considering that oligomeric and polymeric pigments are more resistant to pH changes than the monomeric ones [29] anthocyanin determination is based mainly on free monomeric anthocyanins. Therefore, the decrease observed in total anthocyanin content during maturation is consistent with the participation of monomeric anthocyanins in numerous condensation reactions as well as in hydrolytic and other degradation reactions [30] to a minor extent.

In general, all color parameters were characterized by higher values for Mandilari wines, given their higher anthocyanin content. Chemical age indexes i and ii of Mandilari wines were also higher than the corresponding values measured in Kotsifali, indicating that in Mandilari wines more polymeric pigments are present at wine pH and that those polymeric forms are resistant to SO_2_ bleaching [31].

### 3.2. Statistical Analysis

**Figure 1:**
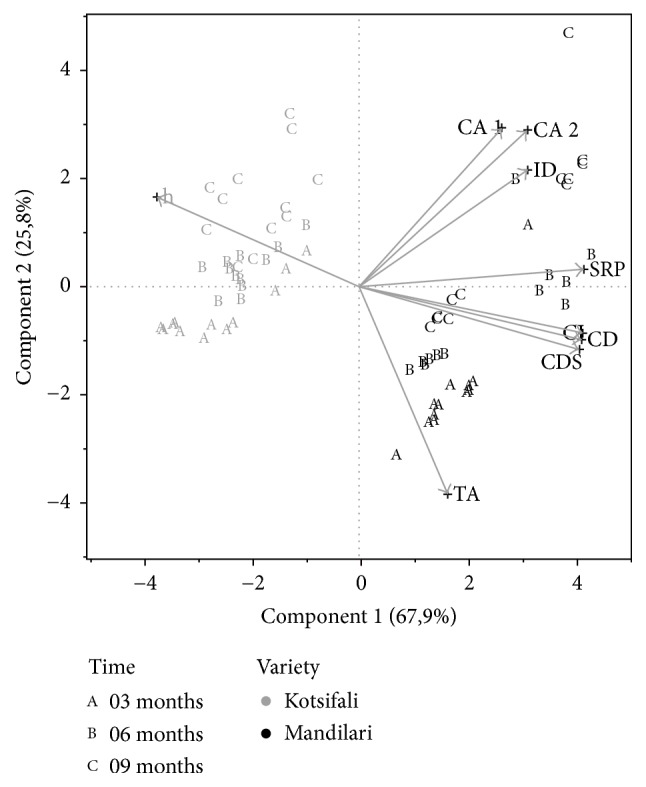
Biplot of principal components 1 and 2 for mean scores of Kotsifali and Mandilari color parameters.

To examine internal structure and patterns of the wine data set Principal Components Analysis was employed. The variables used, namely, total anthocyanins, degree of ionisation of anthocyanins, color density, color density corrected for SO_2_, SO_2_ resistant pigments, chemical ages i and ii, color intensity, and hue, produced the PCA plot shown in Figure 1.

The first two principal components explain 93.7% of the variance. As can be seen in Figure 1, the PCA plot resulted in a clear separation of the two varieties, based on the first principal component. However, a discrimination trend according to maturation time can also be observed, based on the second principal component. Most of the samples that have been aged for 3 months are situated lower than the rest of the samples that have been matured for 6 and 9 months, respectively. Moreover, samples that have been matured for 9 months are mostly found on the top of the diagram. As expected, this trend is more obvious between samples that aged for three months (lower part of the plot) and samples that aged for nine months (higher part of the plot) since it requires more than three months to observe differences in wine chemical parameters.

Given the strong influence of the variety on phenolic content and wine color which is evident in the PCA plot, Analysis of Variance was also performed to examine if any of the variables were independent of variety, vintage, and container effects and were only influenced by time. The results showed that total anthocyanins, hue, pigments resistant to SO_2_, and chemical ages i and ii were influenced by time; however, variety and vintage had a stronger effect in all cases except for chemical age i (Table 3) where time had the most definitive effect.

Table 3 presents the effect tests' results. The Nparm value indicates the number of parameters associated with the effect, DF is the degree of freedom, Sum of Squares gives the Sum of Squares for the hypothesis that the effect is zero, and *F* ratio indicates whether the model differs significantly from a model where all predicted values are the response mean, while the Prob > *F* value measures the probability of obtaining an *F* ratio as large as what is observed. According to Table 2, only the type of container does not have a statistically significant effect on the parameters measured, while the variety exhibits the strongest influence. Variety exhibits the strongest influence on hue (*F* ratio 617.9) followed by SO_2_ resistant pigments (*F* ratio 317.4) and total anthocyanin concentration (*F* ratio 121.1). Vintage seems to have a lesser effect, although in some cases it is more important than time. The only case where time is less dependent on variety and vintage is in chemical age index i (time *F* ratio 61.3 > variety *F* ratio 30.1 > vintage *F* ratio 9).

### 3.3. FT-IR Analysis

According to Analysis of Variance, chemical age index i is the only parameter that is mostly time dependent and can produce significantly statistical differences in the short time period of nine months without strong variety interferences. The conventional analysis employed to measure chemical age indexes i and ii is simple but time consuming and needs chemical agents, making it less appealing to be implemented in the wineries as a routine protocol. When grape, must, or wine is analyzed for quality control, analysis time becomes a key parameter. For this purpose the suitability of FT-IR combined with PLS was investigated as an alternative method to provide information of such technological interest to the wineries.

**Figure 2:**
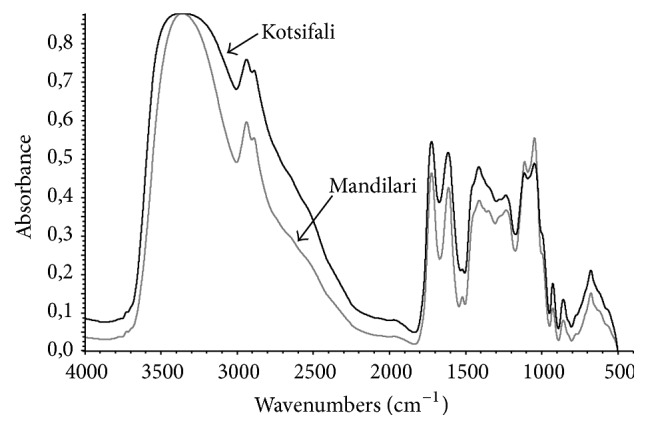
Typical FT-IR spectra for Kotsifali and Mandilari wines.

For this reason, FT-IR coupled with chemometrics was used to develop calibration models. A typical FT-IR spectrum for Kotsifali and Mandilari samples is shown in Figure 2. The region that was selected for further statistical analysis was the region from 1830 to 1500 cm^−1^.

This area as part of the fingerprint region contains much information such as various IR-bands, including those corresponding to the vibrations of the C-O, C-C and C-H bonds [32]. As it is shown in Figure 2, two major peaks are included in the selected area. One centred at 1722 cm^−1^, due to the stretching of carbonyl group (C=O), and one at 1610 cm^−1^, due to C=C stretching (typical for aromatic molecules) [33]. The first peak is mainly connected with the C-O stretching of the esters of hydrolysable tannins, especially derivatives of gallic acid [34] and the C=C stretching, typical of flavanoid-based compounds in wine [35], while the second is mainly attributed to the carboxyl ion (COO-) (symmetrical stretching) [36, 37]. A smaller peak is also detected at 1520 cm^−1^, due to the deformation of the aromatic ring, which can be attributed to simple catechin [11, 35]. The region from 1535 to 1520 cm^−1^ is affected by the structural modifications that occur during polymerization [35] and for this reason it was included in the spectral region used for the statistical analysis. Overall, the selection of the spectral region was based on absorptions that are due to the most important chemical compounds that participate in color stabilization reactions, such as phenolic acids, oak tannins, and flavonoids [7].

Wine spectra are extremely multivariate and hence complex. It is necessary to use advanced mathematical techniques to generate the calibration equations for the individual parameters. This type of calculations is often referred to as chemometrics. The multivariate regression methods have been widely used to provide a better insight into such systems and to build calibration and prediction models. Partial Least Squares (PLS) regression is one of the most used models [38] where the regression algorithm uses the absorptions at selected frequencies or blocks of frequencies to generate an equation that best fits the reference values in a data set. In wine analysis, those models were successfully applied for the determination of various compounds such as anthocyanins and tannins [11, 21], but also ethanol and organic acids (Moreira and Santos, 2005) [39].

**Figure 3:**
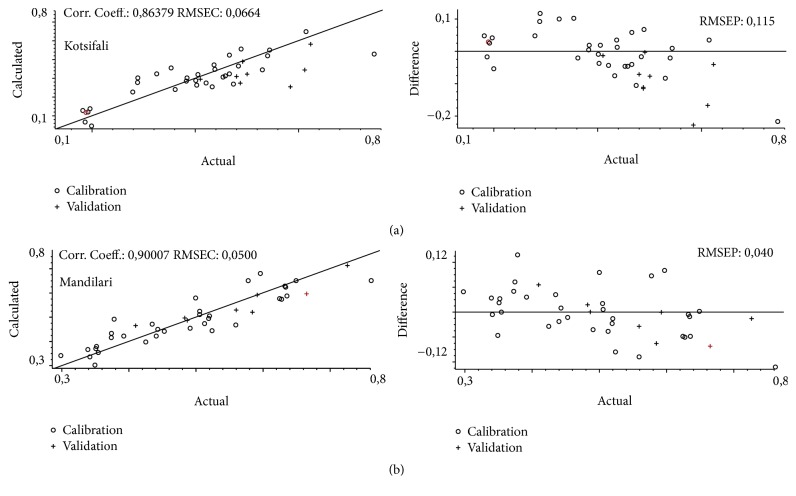
Correlation between the actual chemical age i values and the values predicted by the model for Kotsifali (a) and Mandilari (b) samples.

In this experiment Partial Least Squares were used to develop calibration models for the chemical age indexes. The accuracy of the models is determined by their correlation coefficient (*r*) and the Root Mean Square Error of Calibration (RMSEC) and Root Mean Square Error of Prediction (RMSEP) values. For Kotsifali wines, the models' correlation coefficient (*r*) for chemical age i was 0.86 and the Root Mean Square Error of Calibration (RMSEC) and Root Mean Square Error of Prediction (RMSEP) were 0.066 and 0.115, respectively (Figure 3(a)). For Mandilari samples (Figure 3(b)), the correlation coefficient (*r*) for chemical age i was 0.90 (RMSEC = 0.050, RMSEP = 0.040). For each variety 42 samples were used to build the model, 9 of which were used for validation purposes. For Kotsifali 10 factors were used for each component calculated (Root Mean Square Error of Cross Validation (RMSECV) = 0.164), while for Mandilari 6 were used (RMSECV = 0.089).

**Figure 4:**
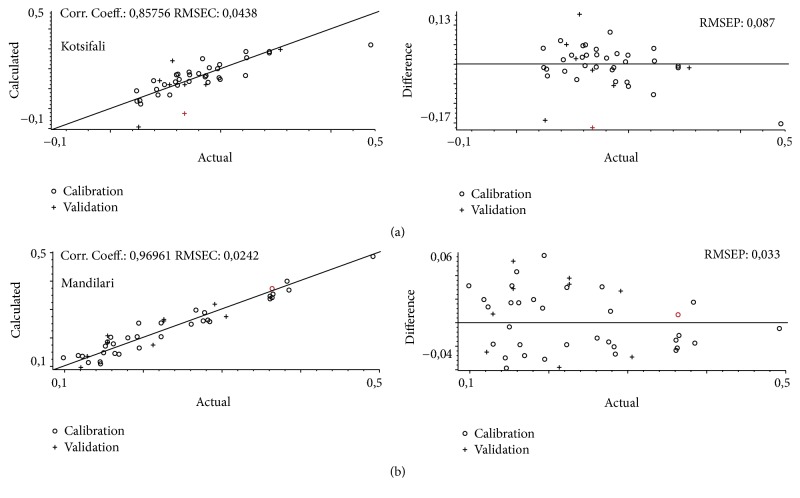
Correlation between the actual chemical age ii values and the values predicted by the model for Kotsifali (a) and Mandilari (b) samples.

For chemical age ii the correlation coefficients (*r*) for Kotsifali and Mandilari wine samples (Figures 4(a) and 4(b)) were 0.86 and 0.97, respectively (Kotsifali: RMSEC = 0.044, RMSEP = 0.088; Mandilari RMSEC = 0.024, RMSEP = 0.033). For each variety 42 samples were used to build the model, 9 of which were used for validation purposes. For both Kotsifali and Mandilari 10 factors were used for each component calculated (Kotsifali RMSECV = 0.214, Mandilari RMSECV = 0.078). Calibration and prediction values for all models as well as Predicted Residual Error Sum of Squares (PRESS) are given as Supplementary Material (Tables 1–4), available online at https://doi.org/10.1155/2017/5767613.

An interesting observation was that, in all cases studied, *r* values of Mandilari were higher than the corresponding values of Kotsifali samples indicating a better fit of the proposed model for wines richer in phenolic compounds including anthocyanins. FT-IR is an indirect analytical method that is product sensitive. Every chemical substance has an IR “fingerprint” that is a function of the molecular bonds that are present in the sample. These characteristic signals are the basis of the measurement and are used in the calibration process. Different grape varieties may contain different compounds with similar IR absorption bands that may interfere with and disturb the calibration model. Indeed, the calibration models developed by FT-IR for musts or wines richer in anthocyanins and acids were more robust compared with those obtained for samples where the concentration of the compounds of interest was lower [13, 22].

In this study, *r* was greater than 0.86 in all cases (higher than 0.90 in Mandilari wines), which is a satisfactory statistical significant value. The closer this value to “one” is, the more linear the relationship between the calculated and actual values is. RMSEC refers to the uncertainty of calibration while RMSEP estimates how well the method should predict concentration values for unknown samples. The calculated RMSEP values for all models were below 0.1, indicating low uncertainty values concerning the methods' prediction ability. The low values of RMSECV also demonstrate that the validation efficiency of the model is satisfactory. These data suggest that chemical age indexes may be estimated using FT-IR and the aforementioned models.

As monomeric anthocyanins decrease during ageing color is no longer determined by monomeric forms; hence there is a lesser need to estimate them individually. In that case polymer forms are of greater importance, for the estimation of which chemical age indexes are more useful. To our knowledge this is the first time FT-IR is used to estimate the chemical age status of a wine.

## 4. Conclusion

Wine ageing has a defining effect on the color of red wines. Chemical age index i values are significantly correlated with maturation time and are less dependent on the variety, the vintage, or the type of container used for maturation. FT-IR combined with PLS allowed developing regression models to provide approximate quantitative values for chemical age indexes in a quick and simple way that can be easily implemented for routine control of wines. Linear relationships were found with correlation coefficients (*r*) 0.93 and 0.91 for the chemical age index i and 0.95 and 0.88 for index ii for Mandilari and Kotsifali samples, respectively. The low RMSEC values in each model demonstrate the robustness of the proposed method. It could be a starting point for the design of more specific models according to the requirements of the wineries.

## Supplementary Material

Actual and predicted values of Chemical age indices (i) and (ii) for Kotsifali and Mandilari samples.

## Figures and Tables

**Table 1:** Samples and containers per vintage (V1: 2012; V2: 2013).

Grape variety	Container	Samples			
		3 months	6 months	9 months	12 months
Kotsifali/Mandilari	Inox tank	V1/V2	V1/V2	V1/V2	V1/—
	Inox tank with oak chips	V1/V2	V1/V2	V1/V2	V1/—
	French oak	V1/V2	V1/V2	V1/V2	V1/—
	American oak	V1/V2	V1/V2	V1/V2	V1/—
	Acacia	V1/V2	V1/V2	V1/V2	V1/—
	Chestnut	V1/V2	V1/V2	V1/V2	V1/—

Sum of samples for both varieties		12/12	12/12	12/12	12/—

**Table 2:** Changes in average values of color parameters and chemical age indexes (CI: color intensity, h: hue, CA1: chemical age i, CA2: chemical age ii, ID: degree of ionisation of anthocyanins, CD: color density, CDS: color density corrected for SO_2_, TA: total anthocyanin concentration, SRP: SO_2_ resistant pigments) during maturation, for Kotsifali and Mandilari wines.

	2012 vintage			2013 vintage		
	3 months	6 months	9 months	3 months	6 months	9 months
Kotsifali						
CI	2.8	3.4	3.9	5.5	5.1	6.1
h	0.9	0.9	1.0	0.7	0.8	0.8
CA 1	0.2	0.4	0.5	0.3	0.4	0.5
CA 2	0.1	0.1	0.2	0.1	0.1	0.2
ID	18.5	15.5	28.3	23.7	23.0	24.8
CD	2.9	4.0	3.2	4.4	4.4	4.5
CDS	3.4	4.5	3.3	4.9	4.5	4.7
TA	143.0	140.5	56.6	145.4	130.7	100.3
SRP	0.4	1.1	0.9	1.0	1.1	1.3
Mandilari						
CI	14.8	16.4	16.2	14.8	12.7	14.0
h	0.5	0.5	0.5	0.5	0.5	0.6
CA 1	0.4	0.6	0.7	0.3	0.4	0.5
CA 2	0.2	0.3	0.4	0.1	0.2	0.2
ID	32.8	34.4	57.4	28.1	26.8	27.7
CD	11.2	13.1	11.6	10.6	10.1	9.9
CDS	12.1	13.3	11.9	11.1	10.2	10.3
TA	285.9	187.8	92.4	322.6	278.4	221.5
SRP	3.2	5.2	4.9	2.6	2.9	3.3

**Table 3:** Test results for fixed effects of hue, chemical age indexes i and ii, total anthocyanins, and SO_2_ resistant pigments for Kotsifali and Mandilari wine samples.

	Nparm	DF	Sum of Squares	*F* ratio	Prob > *F*
*Hue*					
Variety	1	1	2.081079	617.9852	<.0001^*∗*^
Vintage	1	1	0.0592846	17.6048	<.0001^*∗*^
Time	2	2	0.1038602	15.4209	<.0001^*∗*^
Container	5	5	0.0467252	2.775	0.0252^*∗*^
*Chemical age i*					
Variety	1	1	0.15791902	30.0835	<.0001^*∗*^
Vintage	1	1	0.04734925	9.02	0.0038^*∗*^
Time	2	2	0.64369316	61.3116	<.0001^*∗*^
Container	5	5	0.04057336	1.5458	0.1888
*Chemical age ii*					
Variety	1	1	0.13732412	40.686	<.0001^*∗*^
Vintage	1	1	0.05456823	16.1673	0.0002^*∗*^
Time	2	2	0.20179451	29.8935	<.0001^*∗*^
Container	5	5	0.02202906	1.3053	0.2734
*Total anthocyanins*					
Variety	1	1	225840.64	121.065	<.0001^*∗*^
Vintage	1	1	42884.5	22.9888	<.0001^*∗*^
Time	2	2	139047.49	37.2692	<.0001^*∗*^
Container	5	5	8431.81	0.904	0.4843
*SO*_*2*_ * resistant pigments*					
Variety	1	1	135.1368	317.3832	<.0001^*∗*^
Vintage	1	1	6.22457	14.6191	0.0003^*∗*^
Time	2	2	10.32205	12.1212	<.0001^*∗*^
Container	5	5	0.35078	0.1648	0.9745

^*∗*^indicates significant levels.
