# Supplementary material for: Red Wine Age Estimation by the Alteration of Its Color Parameters: Fourier Transform Infrared Spectroscopy as a Tool to Monitor Wine Maturation Time
Source: J Anal Methods Chem. 2017 Nov 1;2017:5767613. doi: 10.1155/2017/5767613 (PMC5687142; doi:10.1155/2017/5767613)
Supplement: Supplementary file 1 — Actual and predicted values of Chemical age indices (i) and (ii) for Kotsifali and Mandilari samples. [file 5767613.f1.pdf]

Table 1. Actual and predicted values of Chemical age index (i) for Kotsifali samples

Sample ID	Usage	Actual	Calculated
1	Calibration	0,189	0,212
2	Validation	0,48	0,431
3	Calibration	0,279	0,315
4	Calibration	0,384	0,331
5	Validation	0,488	0,4
6	Calibration	0,747	0,613
7	Calibration	0,192	0,152
8	Calibration	0,474	0,443
9	Calibration	0,403	0,358
10	Validation	0,501	0,361
11	Validation	0,585	0,369
12	Validation	0,613	0,447
13	Calibration	0,2	0,154
14	Calibration	0,458	0,472
15	Calibration	0,288	0,327
16	Calibration	0,326	0,355
17	Calibration	0,405	0,456
18	Calibration	0,466	0,492
19	Calibration	0,182	0,22
20	Calibration	0,438	0,459
21	Calibration	0,383	0,363
22	Calibration	0,454	0,435
23	Calibration	0,53	0,479
24	Calibration	0,544	0,546
25	Calibration	0,197	0,265
26	Calibration	0,437	0,436
27	Calibration	0,289	0,241
28	Calibration	0,362	0,392
29	Calibration	0,422	0,423
30	Calibration	0,489	0,522
31	Calibration	0,187	0,152
32	Validation	0,41	0,415
33	Calibration	0,354	0,409
34	Calibration	0,433	0,456
35	Validation	0,488	0,384
36	Calibration	0,54	0,555
73	Calibration	0,615	0,63
74	Validation	0,624	0,556
75	Validation	0,492	0,61
76	Calibration	0,484	0,458
77	Calibration	0,466	0,466
78	Calibration	0,401	0,428

Table 2. Actual and predicted values of Chemical age index (i) for Mandilari samples

Sample ID	Usage	Actual	Calculated
37	Calibration	0,52	0,506
38	Calibration	0,762	0,651
39	Calibration	0,352	0,379
40	Calibration	0,435	0,471
41	Calibration	0,524	0,443
42	Calibration	0,634	0,625
43	Calibration	0,298	0,339
44	Calibration	0,597	0,681
45	Calibration	0,349	0,302
46	Calibration	0,453	0,442
47	Calibration	0,513	0,474
48	Validation	0,585	0,522
49	Calibration	0,378	0,493
50	Validation	0,592	0,591
51	Calibration	0,351	0,37
52	Calibration	0,425	0,396
53	Validation	0,483	0,498
54	Calibration	0,519	0,495
55	Calibration	0,374	0,434
56	Calibration	0,578	0,651
57	Calibration	0,354	0,354
58	Calibration	0,443	0,451
59	Calibration	0,491	0,455
60	Calibration	0,506	0,511
61	Calibration	0,373	0,415
62	Calibration	0,559	0,468
63	Calibration	0,341	0,336
64	Validation	0,41	0,465
65	Validation	0,487	0,487
66	Calibration	0,505	0,523
67	Calibration	0,392	0,422
68	Calibration	0,625	0,576
69	Calibration	0,339	0,367
70	Calibration	0,44	0,422
71	Calibration	0,5	0,579
72	Validation	0,56	0,531
79	Validation	0,727	0,715
80	Calibration	0,649	0,651
81	Validation	0,665	0,596
82	Calibration	0,628	0,577
83	Calibration	0,636	0,587
84	Calibration	0,633	0,629

Table 3. Actual and predicted values of Chemical age index (ii) for Kotsifali samples

Sample ID	Usage	Actual	Calculated
1	Validation	0,051	0,027
2	Calibration	0,118	0,135
3	Calibration	0,079	0,138
4	Validation	0,107	0,122
5	Calibration	0,195	0,202
6	Calibration	0,472	0,32
7	Validation	0,052	-0,091
8	Calibration	0,125	0,148
9	Calibration	0,174	0,165
10	Calibration	0,2	0,144
11	Calibration	0,245	0,167
12	Calibration	0,289	0,28
13	Calibration	0,055	0,025
14	Calibration	0,143	0,181
15	Calibration	0,084	0,095
16	Validation	0,09	0,139
17	Calibration	0,12	0,168
18	Calibration	0,168	0,25
19	Calibration	0,048	0,089
20	Calibration	0,122	0,118
21	Calibration	0,143	0,136
22	Calibration	0,161	0,177
23	Calibration	0,198	0,152
24	Calibration	0,247	0,255
25	Calibration	0,054	0,041
26	Validation	0,113	0,24
27	Calibration	0,087	0,069
28	Calibration	0,099	0,122
29	Validation	0,136	0,12
30	Calibration	0,18	0,207
31	Calibration	0,049	0,04
32	Calibration	0,108	0,069
33	Calibration	0,123	0,172
34	Calibration	0,144	0,168
35	Calibration	0,177	0,132
36	Calibration	0,246	0,286
73	Calibration	0,289	0,285
74	Validation	0,308	0,299
75	Calibration	0,199	0,221
76	Validation	0,173	0,119
77	Calibration	0,172	0,157
78	Validation	0,136	-0,025

Table 4. Actual and predicted values of Chemical age index (ii) for Mandilari samples

Sample ID	Usage	Actual	Calculated
37	Validation	0,291	0,318
38	Calibration	0,361	0,337
39	Calibration	0,123	0,136
40	Calibration	0,162	0,179
41	Calibration	0,223	0,204
42	Calibration	0,363	0,341
43	Calibration	0,099	0,13
44	Calibration	0,261	0,248
45	Calibration	0,145	0,115
46	Calibration	0,169	0,142
47	Validation	0,213	0,175
48	Calibration	0,278	0,288
49	Validation	0,155	0,207
50	Validation	0,305	0,276
51	Validation	0,129	0,136
52	Calibration	0,146	0,108
53	Calibration	0,181	0,201
54	Calibration	0,223	0,253
55	Calibration	0,159	0,202
56	Calibration	0,267	0,297
57	Calibration	0,13	0,112
58	Validation	0,155	0,184
59	Calibration	0,195	0,164
60	Validation	0,226	0,259
61	Calibration	0,154	0,185
62	Calibration	0,276	0,26
63	Validation	0,121	0,096
64	Calibration	0,152	0,169
65	Calibration	0,192	0,204
66	Validation	0,226	0,264
67	Calibration	0,164	0,145
68	Calibration	0,284	0,257
69	Calibration	0,118	0,137
70	Calibration	0,15	0,147
71	Calibration	0,194	0,251
72	Calibration	0,282	0,262
79	Calibration	0,492	0,487
80	Calibration	0,383	0,4
81	Calibration	0,385	0,367
82	Calibration	0,361	0,346
83	Calibration	0,365	0,354
84	Calibration	0,364	0,371

Figure 1. Predicted Residual Error Sum of Squares (PRESS) values and Root Mean Square Error of Validation (RMSECV) for each factor for Kotsifali Chemical age (i) prediction.

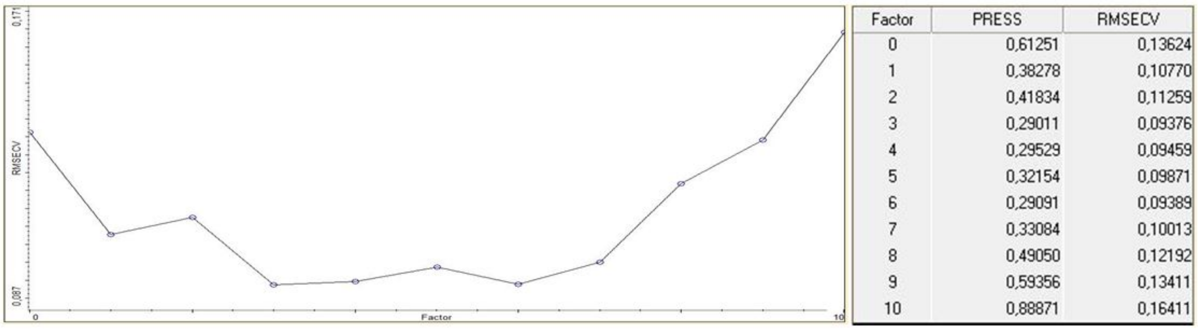

Factor	PRESS	RMSECV
0	0,61251	0,13624
1	0,38278	0,10770
2	0,41834	0,11259
3	0,29011	0,09376
4	0,29529	0,09459
5	0,32154	0,09871
6	0,29091	0,09389
7	0,33084	0,10013
8	0,49050	0,12192
9	0,59356	0,13411
10	0,88871	0,16411

Figure 2. . Predicted Residual Error Sum of Squares (PRESS) values and Root Mean Square Error of Validation (RMSECV) for each factor for Mandilari Chemical age (i) prediction.

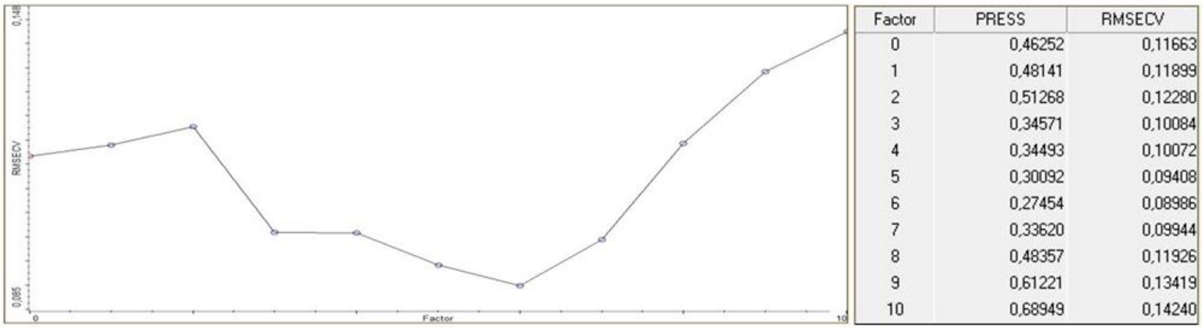

Factor	PRESS	RMSECV
0	0,46252	0,11663
1	0,48141	0,11899
2	0,51268	0,12280
3	0,34571	0,10084
4	0,34493	0,10072
5	0,30092	0,09408
6	0,27454	0,08986
7	0,33620	0,09944
8	0,48357	0,11926
9	0,61221	0,13419
10	0,68949	0,14240

Figure 3. Predicted Residual Error Sum of Squares (PRESS) values and Root Mean Square Error of Validation (RMSECV) for each factor for Kotsifali Chemical age (ii) prediction.

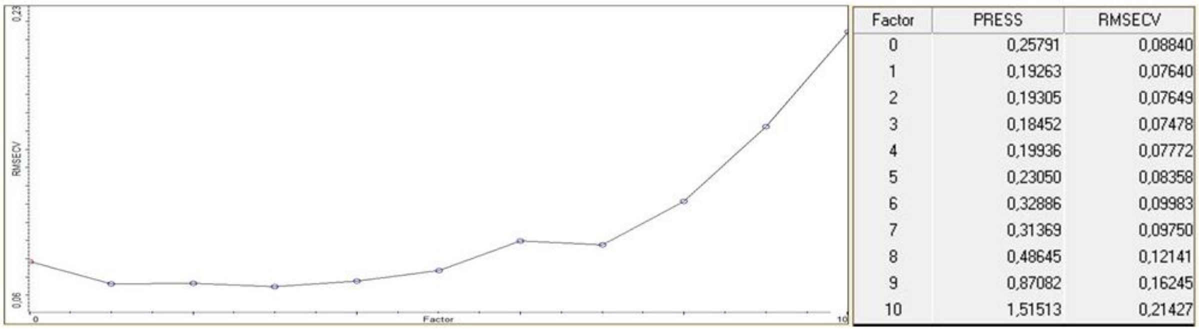

Factor	PRESS	RMSECV
0	0,25791	0,08840
1	0,19263	0,07640
2	0,19305	0,07649
3	0,18452	0,07478
4	0,19936	0,07772
5	0,23050	0,08358
6	0,32886	0,09983
7	0,31369	0,09750
8	0,48645	0,12141
9	0,87082	0,16245
10	1,51513	0,21427

Figure 4. . Predicted Residual Error Sum of Squares (PRESS) values and Root Mean Square Error of Validation (RMSECV) for each factor for Mandilari Chemical age (ii) prediction.

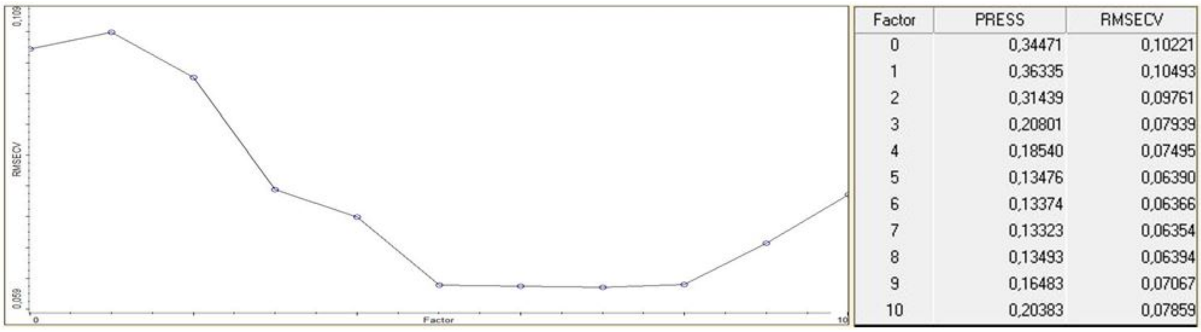

Factor	PRESS	RMSECV
0	0,34471	0,10221
1	0,36335	0,10493
2	0,31439	0,09761
3	0,20801	0,07939
4	0,18540	0,07495
5	0,13476	0,06390
6	0,13374	0,06366
7	0,13323	0,06354
8	0,13493	0,06394
9	0,16483	0,07067
10	0,20383	0,07859
